# Supplementary material for: No Transcriptional Compensation for Extreme Gene Dosage Imbalance in Fragmented Bacterial Endosymbionts of Cicadas
Source: Genome Biol Evol. 2023 Jun 2;15(6):evad100. doi: 10.1093/gbe/evad100 (PMC10287537; doi:10.1093/gbe/evad100)
Supplement: evad100_Supplementary_Data [file evad100_supplementary_data.pdf]

No Transcriptional Compensation for Extreme Gene Dosage Imbalance in Fragmented Bacterial Endosymbionts of Cicadas

Noah Spencer, Piotr Łukasik, Mariah Meyer, Claudio Veloso and John P. McCutcheon

April 2023

Supplementary Material

Supplementary Table S1 | *Hodgkinia* genome complexity and abundance ratios from each specimen analyzed. Relative abundances are given for each biological replicate (labeled A, B, and C) of a given cicada species. A relative abundance value of "X" for any genome/sample combination indicates missing data.

Insect Host	Genome/Contig Name (Accession)	Number of Functional Genes	Length (bp)	Relative Abundance (A)	Relative Abundance (B)	Relative Abundance (C)
Diceroprocta near semicincta	DB174 (CP118773.1)	162	143746	1	1	1
Tettigades ulnaria	TETULN (CP008699.1)	193	150297	1	1	**X**
Tettigades undata	TETUND1 (CP007232.1)	138	133698	0.42	0.59	0.45
Tettigades undata	TETUND2 (CP007233.1)	165	140570	0.58	0.41	0.55
Okanagana oregona	OKAORE1 (CP119775.1)	74	130417	0.41	0.29	0.38
Okanagana oregona	OKAORE2 (CP119777.1)	60	121230	0.17	0.23	0.12
Okanagana oregona	OKAORE3 (CP119776.1)	58	119979	0.02	0.02	0.02
Okanagana oregona	OKAORE4 (CP119778.1)	132	111516	0.4	0.47	0.48
Tettigades limbata	TETLIM1 (CP024748.1)	149	145143	0.75	0.73	0.73
Tettigades limbata	TETLIM2 (CP024747.1)	72	130881	0.1	0.12	0.12
Tettigades limbata	TETLIM3 (CP024746.1)	43	128037	0.01	0.06	0.06
Tettigades limbata	TETLIM4 (CP024745.1)	45	126271	0.09	0.08	0.08
Tettigades limbata	TETLIM5 (CP024744.1)	39	121924	0.04	0.01	0.01

Continued on next page

Supplementary Table S1 – continued from previous page

Insect Host	Genome/Contig Name (Accession)	Number of Functional Genes	Length (bp)	Relative Abundance (A)	Relative Abundance (B)	Relative Abundance (C)
Magicicada septendecim	CM008730.1	22	63626	0.07	**X**	0.04
Magicicada septendecim	CM008741.1	7	61848	0.03	**X**	0.05
Magicicada septendecim	CM008752.1	7	61285	0.01	**X**	0.02
Magicicada septendecim	CM008763.1	20	57162	0.09	**X**	0.08
Magicicada septendecim	CM008764.1	12	56298	0.05	**X**	0.02
Magicicada septendecim	CM008765.1	9	56278	0.01	**X**	0.02
Magicicada septendecim	CM008766.1	12	48066	0.04	**X**	0.02
Magicicada septendecim	CM008767.1	3	43440	0	**X**	0
Magicicada septendecim	CM008768.1	4	34489	0.01	**X**	0.02
Magicicada septendecim	CM008731.1	3	33034	0.01	**X**	0.02
Magicicada septendecim	CM008732.1	4	28244	0.01	**X**	0.01
Magicicada septendecim	CM008733.1	5	22960	0	**X**	0.01
Magicicada septendecim	CM008734.1	4	22204	0.01	**X**	0.01
Magicicada septendecim	CM008735.1	3	21903	0.01	**X**	0.01
Magicicada septendecim	CM008736.1	2	21496	0.01	**X**	0.01
Magicicada septendecim	CM008737.1	2	19133	0	**X**	0.01
Magicicada septendecim	CM008738.1	4	18932	0	**X**	0
Magicicada septendecim	CM008739.1	1	17726	0	**X**	0
Magicicada septendecim	CM008740.1	3	15979	0.01	**X**	0.01
Magicicada septendecim	CM008742.1	2	11397	0	**X**	0
Magicicada septendecim	CM008743.1	2	11166	0	**X**	0.01
Magicicada septendecim	CM008744.1	2	3224	0	**X**	0
Magicicada septendecim	CM008745.1	1	1099	0	**X**	0
Magicicada septendecim	CM008746.1	4	49806	0.02	**X**	0.03
Magicicada septendecim	CM008747.1	19	42010	0.09	**X**	0.07
Magicicada septendecim	CM008748.1	11	33162	0.04	**X**	0.03

Continued on next page

Supplementary Table S1 – continued from previous page

Insect Host	Genome/Contig Name (Accession)	Number of Functional Genes	Length (bp)	Relative Abundance (A)	Relative Abundance (B)	Relative Abundance (C)
Magicicada septendecim	CM008749.1	5	30710	0.02	**X**	0.03
Magicicada septendecim	CM008750.1	6	30380	0	**X**	0
Magicicada septendecim	CM008751.1	3	26344	0.01	**X**	0.01
Magicicada septendecim	CM008753.1	5	23491	0.06	**X**	0.04
Magicicada septendecim	CM008754.1	5	23305	0	**X**	0.01
Magicicada septendecim	CM008755.1	4	20205	0.02	**X**	0.01
Magicicada septendecim	CM008756.1	4	19057	0	**X**	0.01
Magicicada septendecim	CM008757.1	3	19055	0	**X**	0
Magicicada septendecim	CM008758.1	3	16038	0.01	**X**	0.01
Magicicada septendecim	CM008759.1	3	14776	0	**X**	0
Magicicada septendecim	CM008760.1	6	13724	0.01	**X**	0.01
Magicicada septendecim	CM008761.1	3	10218	0.01	**X**	0.01
Magicicada septendecim	CM008762.1	2	10207	0	**X**	0
Magicicada septendecim	NXGN01000045.1	9	55416	0.01	**X**	0.01
Magicicada septendecim	NXGN01000040.1	0	1242	0	**X**	0
Magicicada septendecim	NXGN01000041.1	0	1228	0	**X**	0
Magicicada septendecim	NXGN01000042.1	0	2577	0.01	**X**	0.01
Magicicada septendecim	NXGN01000043.1	1	1223	0	**X**	0
Magicicada septendecim	NXGN01000044.1	0	2556	0	**X**	0
Magicicada septendecim	NXGN01000046.1	0	2541	0.01	**X**	0
Magicicada septendecim	NXGN01000047.1	1	7353	0	**X**	0
Magicicada septendecim	NXGN01000048.1	4	7344	0	**X**	0
Magicicada septendecim	NXGN01000049.1	0	7108	0	**X**	0
Magicicada septendecim	NXGN01000050.1	0	6995	0.02	**X**	0.02
Magicicada septendecim	NXGN01000051.1	0	2330	0	**X**	0
Magicicada septendecim	NXGN01000052.1	0	6877	0.01	**X**	0.01

Continued on next page

Supplementary Table S1 – continued from previous page

Insect Host	Genome/Contig Name (Accession)	Number of Functional Genes	Length (bp)	Relative Abundance (A)	Relative Abundance (B)	Relative Abundance (C)
Magicicada septendecim	NXGN01000053.1	0	6719	0	**X**	0
Magicicada septendecim	NXGN01000054.1	0	1136	0	**X**	0
Magicicada septendecim	NXGN01000055.1	1	1126	0	**X**	0
Magicicada septendecim	NXGN01000056.1	1	6536	0	**X**	0
Magicicada septendecim	NXGN01000057.1	0	1095	0	**X**	0
Magicicada septendecim	NXGN01000058.1	0	1074	0	**X**	0
Magicicada septendecim	NXGN01000059.1	2	6172	0	**X**	0.01
Magicicada septendecim	NXGN01000060.1	0	1067	0	**X**	0
Magicicada septendecim	NXGN01000061.1	1	1988	0	**X**	0
Magicicada septendecim	NXGN01000062.1	0	1986	0	**X**	0
Magicicada septendecim	NXGN01000063.1	0	1060	0	**X**	0
Magicicada septendecim	NXGN01000064.1	0	1055	0	**X**	0
Magicicada septendecim	NXGN01000093.1	16	93841	0.03	**X**	0.04
Magicicada septendecim	NXGN01000066.1	2	13240	0	**X**	0
Magicicada septendecim	NXGN01000065.1	0	1052	0	**X**	0
Magicicada septendecim	NXGN01000068.1	5	13161	0.01	**X**	0.01
Magicicada septendecim	NXGN01000067.1	0	1049	0	**X**	0
Magicicada septendecim	NXGN01000069.1	1	1924	0.01	**X**	0.01
Magicicada septendecim	NXGN01000070.1	0	1039	0	**X**	0
Magicicada septendecim	NXGN01000072.1	0	13003	0	**X**	0
Magicicada septendecim	NXGN01000071.1	0	1034	0	**X**	0
Magicicada septendecim	NXGN01000073.1	0	1033	0	**X**	0
Magicicada septendecim	NXGN01000074.1	1	1893	0.01	**X**	0.01
Magicicada septendecim	NXGN01000075.1	0	1029	0	**X**	0
Magicicada septendecim	NXGN01000076.1	1	5800	0	**X**	0.01
Magicicada septendecim	NXGN01000077.1	1	1865	0.01	**X**	0

Continued on next page

Supplementary Table S1 – continued from previous page

Insect Host	Genome/Contig Name (Accession)	Number of Functional Genes	Length (bp)	Relative Abundance (A)	Relative Abundance (B)	Relative Abundance (C)
Magicicada septendecim	NXGN01000078.1	0	1012	0	**X**	0
Magicicada septendecim	NXGN01000079.1	2	5637	0.02	**X**	0.02
Magicicada septendecim	NXGN01000080.1	0	1007	0	**X**	0
Magicicada septendecim	NXGN01000081.1	0	1004	0	**X**	0
Magicicada septendecim	NXGN01000082.1	0	12440	0.01	**X**	0.01
Magicicada septendecim	NXGN01000083.1	1	993	0	**X**	0
Magicicada septendecim	NXGN01000084.1	0	991	0	**X**	0
Magicicada septendecim	NXGN01000085.1	0	989	0	**X**	0
Magicicada septendecim	NXGN01000086.1	0	5483	0.02	**X**	0.02
Magicicada septendecim	NXGN01000087.1	1	987	0	**X**	0
Magicicada septendecim	NXGN01000088.1	1	5409	0.01	**X**	0.01
Magicicada septendecim	NXGN01000089.1	0	976	0	**X**	0
Magicicada septendecim	NXGN01000090.1	1	1756	0	**X**	0
Magicicada septendecim	NXGN01000091.1	0	954	0	**X**	0
Magicicada septendecim	NXGN01000092.1	1	1715	0	**X**	0
Magicicada septendecim	NXGN01000104.1	7	61285	0.01	**X**	0.02
Magicicada septendecim	NXGN01000094.1	0	947	0	**X**	0
Magicicada septendecim	NXGN01000095.1	3	1697	0	**X**	0
Magicicada septendecim	NXGN01000096.1	1	938	0	**X**	0
Magicicada septendecim	NXGN01000097.1	0	926	0	**X**	0
Magicicada septendecim	NXGN01000098.1	0	926	0	**X**	0
Magicicada septendecim	NXGN01000099.1	0	917	0	**X**	0
Magicicada septendecim	NXGN01000100.1	0	911	0	**X**	0
Magicicada septendecim	NXGN01000101.1	0	895	0	**X**	0
Magicicada septendecim	NXGN01000102.1	2	10545	0	**X**	0
Magicicada septendecim	NXGN01000103.1	0	879	0	**X**	0

Continued on next page

Supplementary Table S1 – continued from previous page

Insect Host	Genome/Contig Name (Accession)	Number of Functional Genes	Length (bp)	Relative Abundance (A)	Relative Abundance (B)	Relative Abundance (C)
Magicicada septendecim	NXGN01000105.1	0	875	0	**X**	0
Magicicada septendecim	NXGN01000106.1	0	875	0	**X**	0
Magicicada septendecim	NXGN01000107.1	0	874	0	**X**	0
Magicicada septendecim	NXGN01000108.1	1	867	0	**X**	0
Magicicada septendecim	NXGN01000109.1	0	1551	0	**X**	0
Magicicada septendecim	NXGN01000110.1	0	1549	0	**X**	0
Magicicada septendecim	NXGN01000111.1	0	859	0	**X**	0
Magicicada septendecim	NXGN01000112.1	0	857	0	**X**	0
Magicicada septendecim	NXGN01000113.1	0	4285	0	**X**	0.01
Magicicada septendecim	NXGN01000114.1	0	853	0	**X**	0
Magicicada septendecim	NXGN01000115.1	0	1521	0	**X**	0
Magicicada septendecim	NXGN01000116.1	0	847	0	**X**	0
Magicicada septendecim	NXGN01000117.1	0	1511	0	**X**	0
Magicicada septendecim	NXGN01000118.1	0	841	0	**X**	0
Magicicada septendecim	NXGN01000119.1	0	1509	0	**X**	0
Magicicada septendecim	NXGN01000120.1	0	837	0	**X**	0
Magicicada septendecim	NXGN01000121.1	0	836	0	**X**	0
Magicicada septendecim	NXGN01000122.1	0	831	0	**X**	0
Magicicada septendecim	NXGN01000123.1	0	1491	0	**X**	0
Magicicada septendecim	NXGN01000124.1	0	823	0	**X**	0
Magicicada septendecim	NXGN01000125.1	0	817	0	**X**	0
Magicicada septendecim	NXGN01000126.1	1	9692	0	**X**	0.01
Magicicada septendecim	NXGN01000127.1	0	809	0	**X**	0
Magicicada septendecim	NXGN01000128.1	0	1438	0	**X**	0
Magicicada septendecim	NXGN01000129.1	0	794	0	**X**	0
Magicicada septendecim	NXGN01000130.1	0	793	0	**X**	0

Continued on next page

Supplementary Table S1 – continued from previous page

Insect Host	Genome/Contig Name (Accession)	Number of Functional Genes	Length (bp)	Relative Abundance (A)	Relative Abundance (B)	Relative Abundance (C)
Magicicada septendecim	NXGN01000131.1	0	790	0	**X**	0
Magicicada septendecim	NXGN01000132.1	3	3640	0	**X**	0
Magicicada septendecim	NXGN01000133.1	0	779	0	**X**	0
Magicicada septendecim	NXGN01000134.1	0	779	0	**X**	0
Magicicada septendecim	NXGN01000135.1	0	777	0	**X**	0
Magicicada septendecim	NXGN01000136.1	1	1403	0	**X**	0
Magicicada septendecim	NXGN01000137.1	1	3491	0	**X**	0
Magicicada septendecim	NXGN01000138.1	0	1389	0	**X**	0
Magicicada septendecim	NXGN01000139.1	0	764	0	**X**	0
Magicicada septendecim	NXGN01000140.1	3	3415	0.01	**X**	0.01
Magicicada septendecim	NXGN01000141.1	0	761	0	**X**	0
Magicicada septendecim	NXGN01000142.1	0	759	0	**X**	0
Magicicada septendecim	NXGN01000143.1	2	8842	0	**X**	0.01
Magicicada septendecim	NXGN01000144.1	0	1371	0	**X**	0
Magicicada septendecim	NXGN01000145.1	0	754	0	**X**	0
Magicicada septendecim	NXGN01000146.1	0	752	0	**X**	0
Magicicada septendecim	NXGN01000147.1	0	751	0	**X**	0
Magicicada septendecim	NXGN01000148.1	0	737	0	**X**	0
Magicicada septendecim	NXGN01000149.1	1	3175	0	**X**	0
Magicicada septendecim	NXGN01000150.1	0	580	0	**X**	0
Magicicada septendecim	NXGN01000151.1	0	492	0	**X**	0
Magicicada septendecim	NXGN01000152.1	0	489	0	**X**	0
Magicicada septendecim	NXGN01000153.1	0	478	0	**X**	0
Magicicada septendecim	NXGN01000154.1	0	473	0	**X**	0
Magicicada septendecim	NXGN01000155.1	3	2962	0.01	**X**	0.01
Magicicada septendecim	NXGN01000156.1	0	8187	0	**X**	0

Continued on next page

Supplementary Table S1 – continued from previous page

Insect Host	Genome/Contig Name (Accession)	Number of Functional Genes	Length (bp)	Relative Abundance (A)	Relative Abundance (B)	Relative Abundance (C)
Magicicada septendecim	NXGN01000157.1	0	2888	0	**X**	0
Magicicada septendecim	NXGN01000158.1	1	2885	0	**X**	0
Magicicada septendecim	NXGN01000159.1	0	2873	0	**X**	0
Magicicada septendecim	NXGN01000160.1	1	2853	0.01	**X**	0
Magicicada septendecim	NXGN01000161.1	1	7883	0	**X**	0
Magicicada septendecim	NXGN01000162.1	0	2705	0	**X**	0
Magicicada septendecim	NXGN01000163.1	3	7735	0.02	**X**	0.02

Supplementary Table S2 | Antisense counts as a percentage of sense+antisense counts at functional genes in *Hodgkinia* and *Sulcia* genomes from each specimen analyzed. Values of "X" indicate missing data.

Cicada species	Specimen	% Antisense *Hodgkinia* Transcription	% Antisense *Sulcia* Transcription
Diceroprocta near *semicincta*	A	48.9%	15.5%
Diceroprocta near *semicincta*	B	43.8%	13.0%
Diceroprocta near *semicincta*	C	46.4%	15.6%
Tettigades ulnaria	A	22.8%	15.8%
Tettigades ulnaria	B	35%	18.0%
Tettigades ulnaria	C	**X**	22.3%
Tettigades undata	A	26.2%	20.6%
Tettigades undata	B	35.2%	30.6%
Tettigades undata	C	30.3%	26.0%
Okanagana oregona	A	25.8%	23.3%
Okanagana oregona	B	35.7%	22.7%
Okanagana oregona	C	28.4%	22.3%
Tettigades limbata	A	25.7%	16.4%
Tettigades limbata	B	31.6%	13.6%
Tettigades limbata	C	36.7%	16.6%
Magicicada septendecim	A	12+	11.4%
Magicicada septendecim	B	**X**	**X**
Magicicada septendecim	C	12+	13.8%

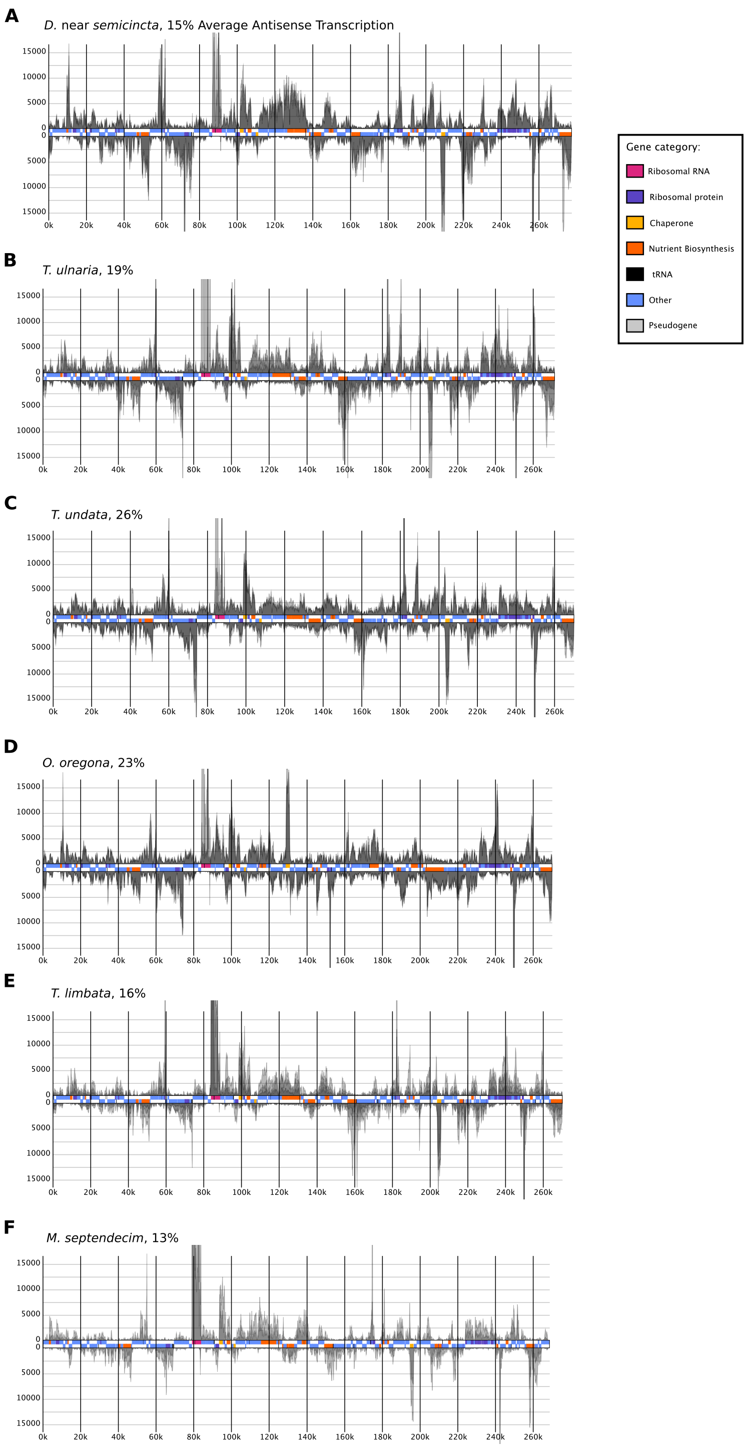

Supplementary Figure S1: Sulcia RNA coverage plots and percent antisense transcription from all cicada species examined. *Rectangles in the central track of each plot represent annotated genes and are colored according to functional categories. Positive and negative Y axes correspond to coverage of unfiltered RNA-seq reads derived from the plus and minus strands of each chromosome, respectively. Coverage represents alignments downsampled to approximately 3500X mean coverage of each genome and is cropped at approximately* $y=\pm 20000$. *Antisense countsas a percentage of sense+antisense counts (averaged across biological replicates) for each Sulcia lineage are shown next to the name of the corresponding host species.*

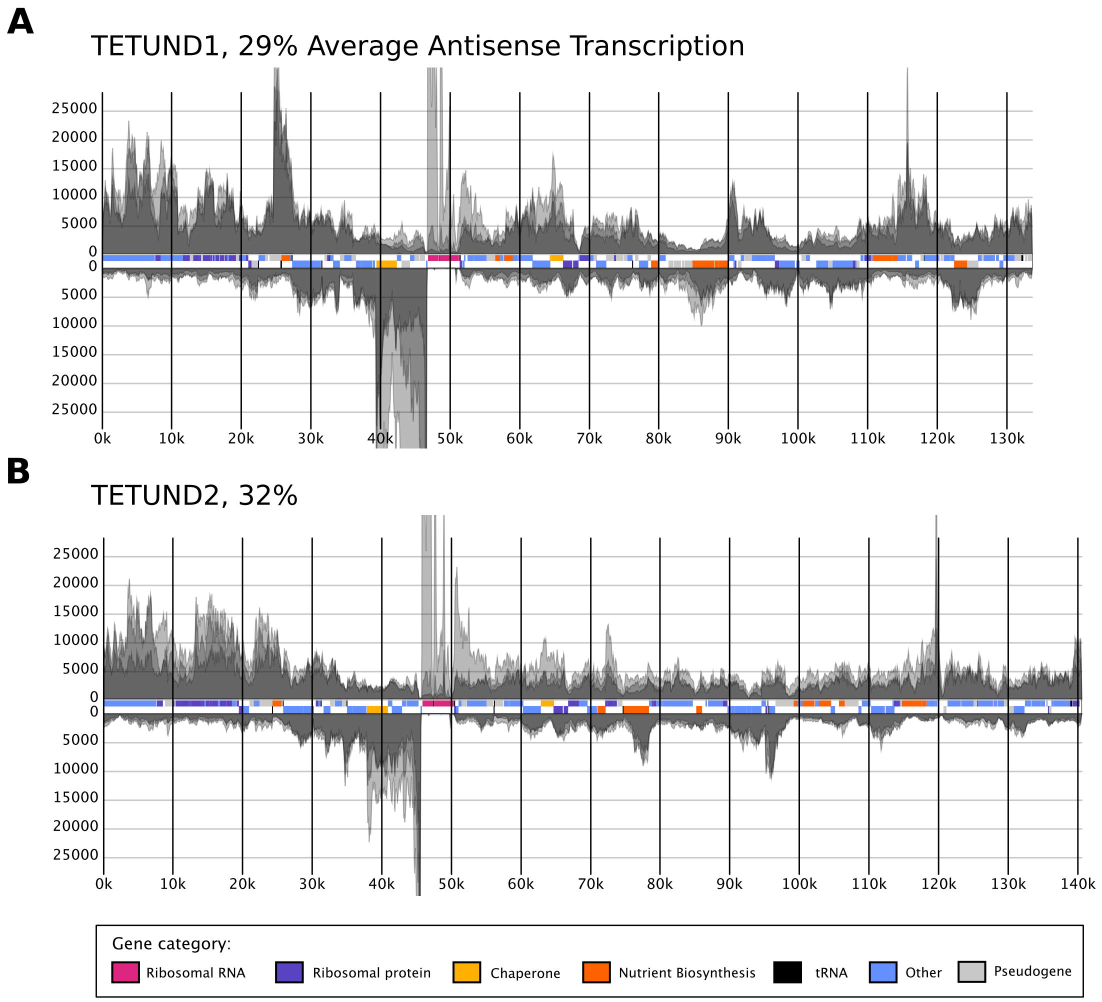

Supplementary Figure S2: RNA coverage plots from all genomic lineages of Hodgkinia present in Tettigades undata. *Rectangles in the central track of each plot represent annotated genes and are colored according to functional categories. Positive and negative Y axes correspond to coverage of unfiltered RNA-seq reads derived from the plus and minus strands of each chromosome, respectively and are cropped at approximately y=±30000. Antisense counts are shown as a percentage of sense+antisense counts (averaged across biological replicates) for each lineage.*

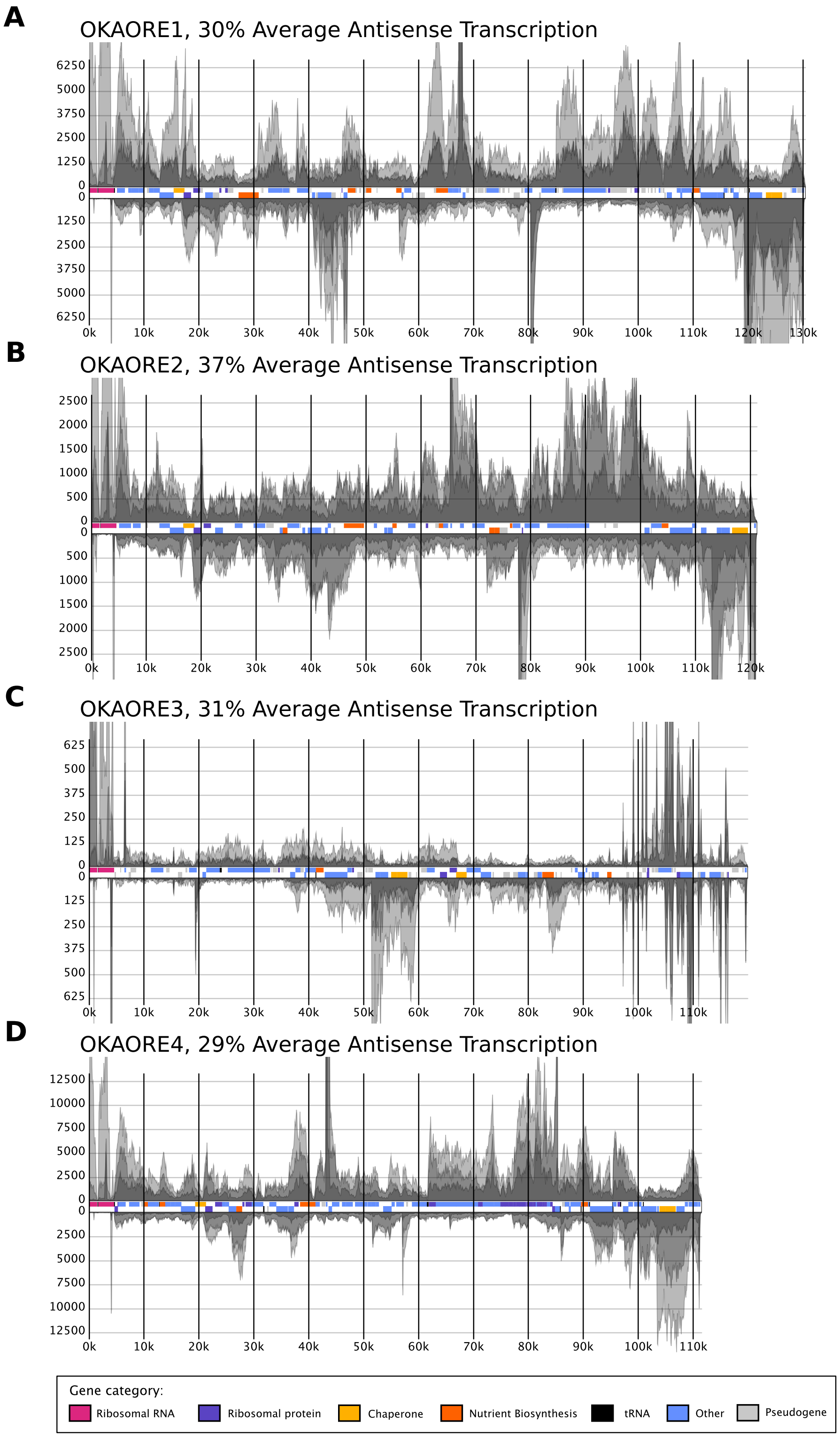

Supplementary Figure S3: RNA coverage plots from all genomic lineages of Hodgkinia present in Okanagana oregona. *Rectangles in the central track of each plot represent annotated genes and are colored according to functional categories. Positive and negative Y axes correspond to coverage of unfiltered RNA-seq reads derived from the plus and minus strands of each chromosome respectively and are cropped for readability. Antisense counts are shown as a percentage of sense+antisense counts (averaged across biological replicates) for each lineage.*

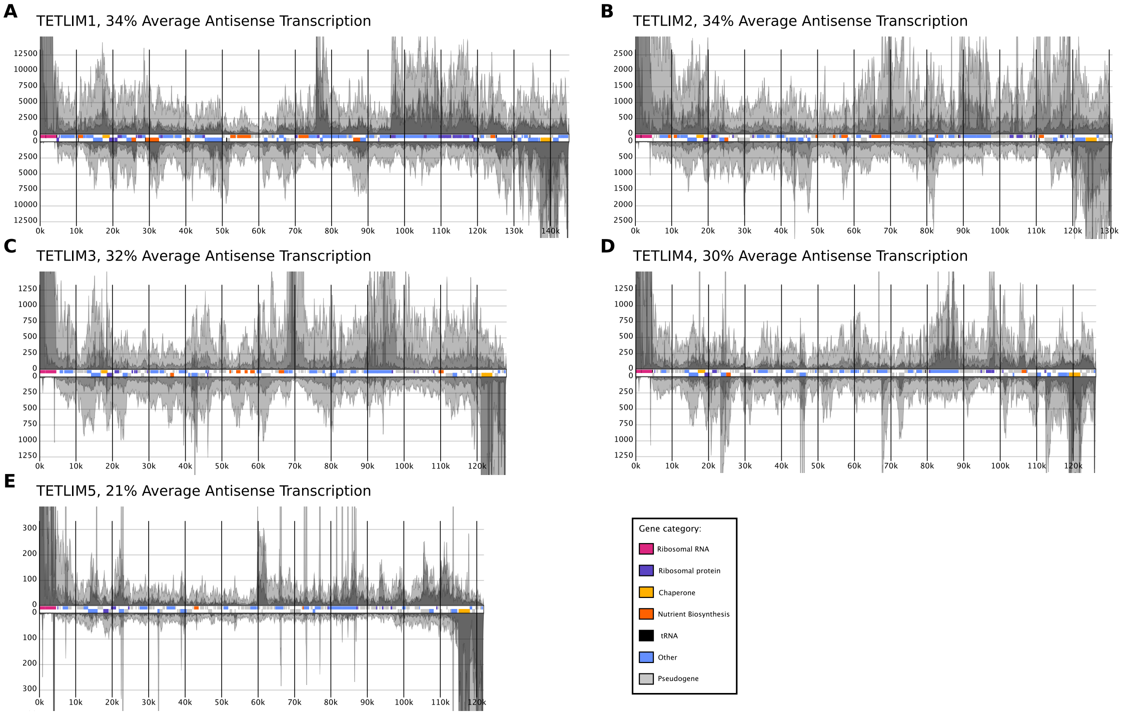

Supplementary Figure S4: RNA coverage plots from all genomic lineages of Hodgkinia present in Tettigades limbata. *Rectangles in the central track of each plot represent annotated genes and are colored according to functional categories. Positive and negative Y axes correspond to coverage of unfiltered RNA-seq reads derived from the plus and minus strands of each chromosome respectively and are cropped for readability. Antisense counts are shown as a percentage of sense+antisense counts (averaged across biological replicates) for each lineage.*

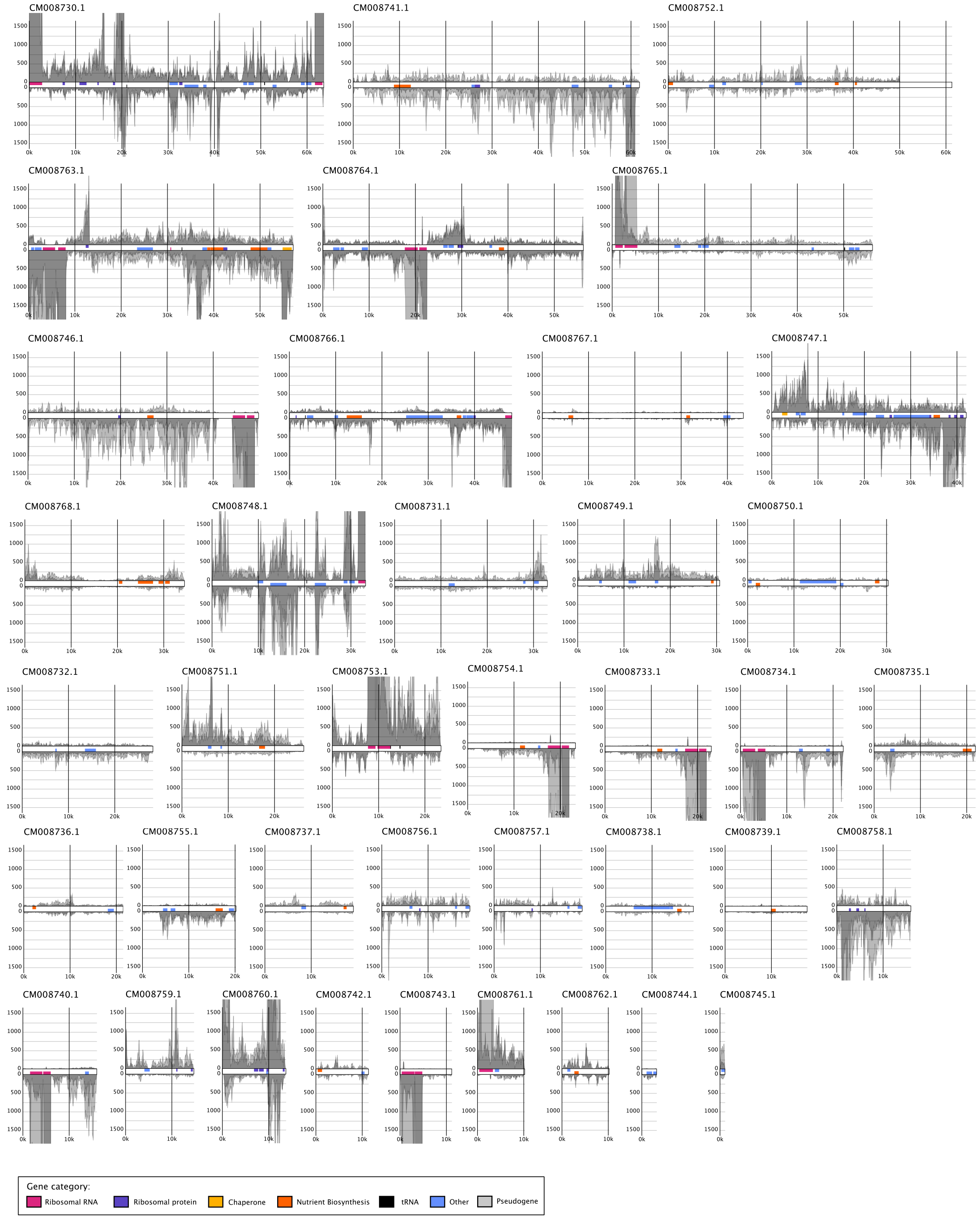

Supplementary Figure S5: RNA coverage plots of all complete Hodgkinia genomic circles present in Magicicada septendecim. *Rectangles in the central track of each plot represent annotated genes and are colored according to functional categories. Positive and negative Y axes correspond to coverage of unfiltered RNA-seq reads derived from the plus and minus strands of each chromosome respectively and are cropped for readability. Plots are ordered by contig length. On average, these Hodgkinia exhibited 26% antisense counts.*

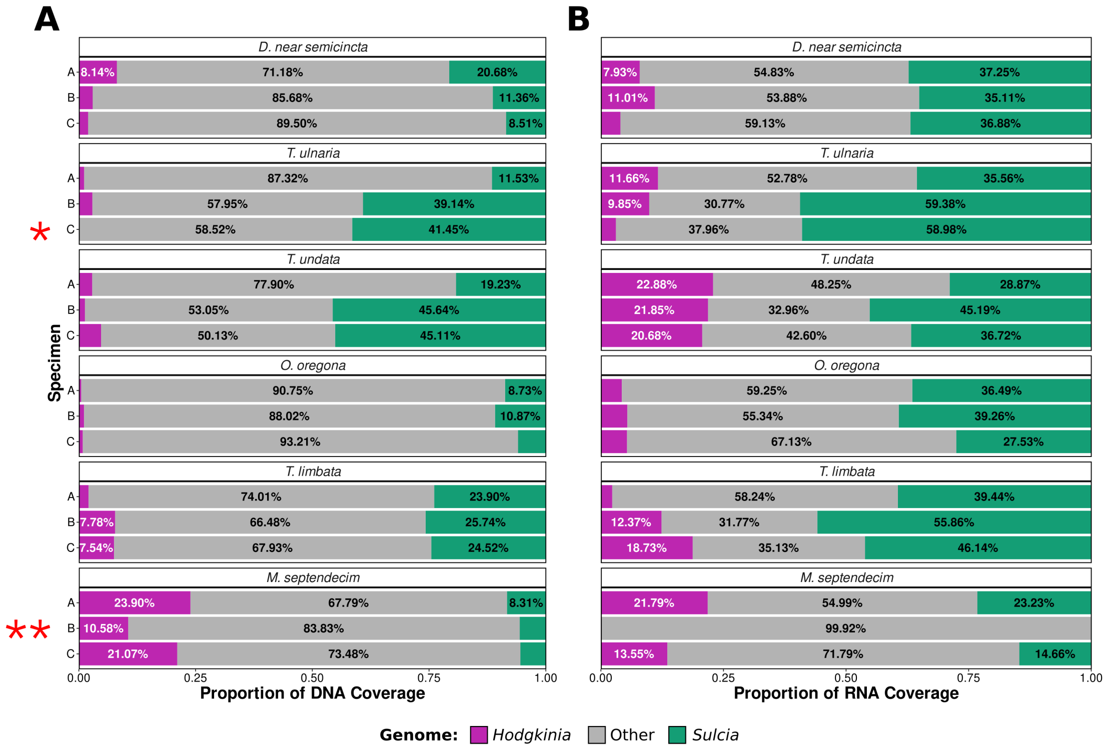

Supplementary Figure S6: Contributions of Hodgkinia and Sulcia to DNA and RNA sequencing coverage prior to bioinformatic rRNA sequence removal. (A) *DNA and* ***(B)*** *RNA sequencing coverage of Hodgkinia (magenta) and Sulcia (green) genomes as a percentage of total reads in triplicate biological replicates for each of the six cicada species examined. T. ulnaria specimen C (one asterisk) was excluded from Hodgkinia-based analyses. M. septendecim specimen B (two asterisks) was excluded from all further analyses.*

A

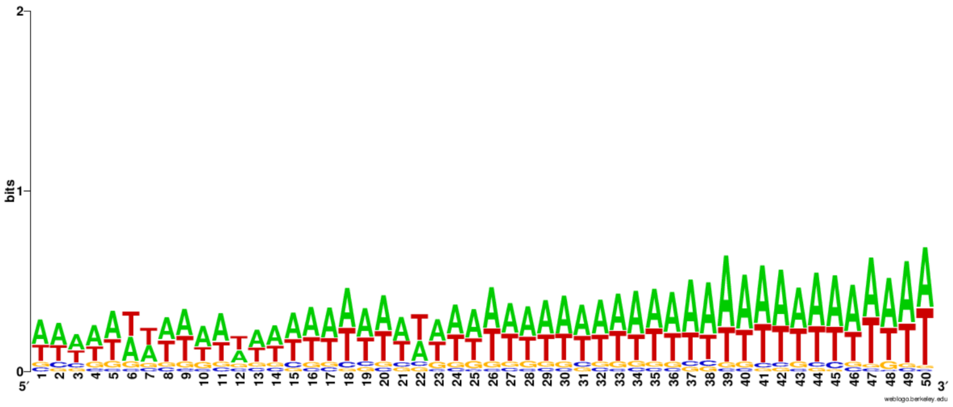

B

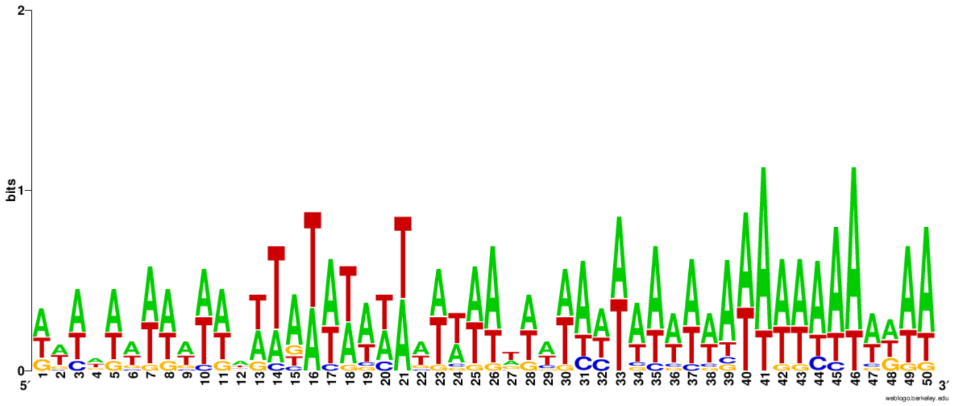

C

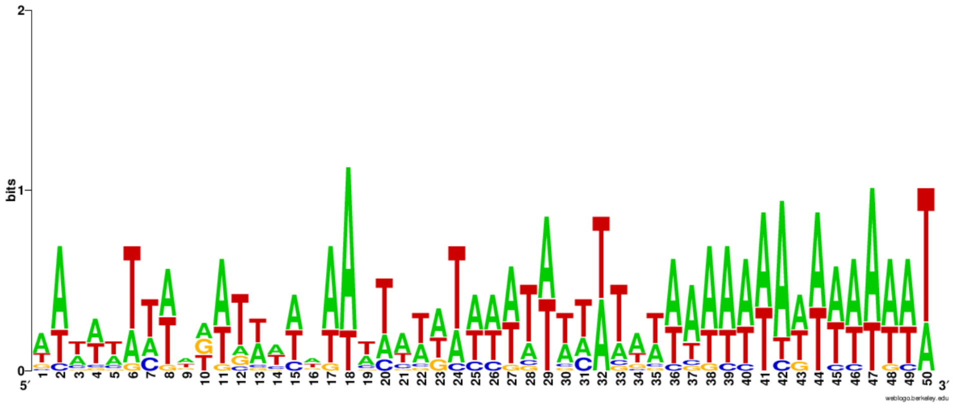

Supplementary Figure S7: Logo plots of sequences immediately upstream from start codons in Sulcia from Tettigades ulnaria. *Sequences were retrieved from either **(A)** all protein-coding genes, **(B)** the fifteen protein-coding genes with the highest average expression levels, or **(C)** the fifteen protein-coding genes with the lowest average expression levels. In each case, A and T bases are highly represented, which simply reflects the AT-rich nature of all Sulcia genomes.*

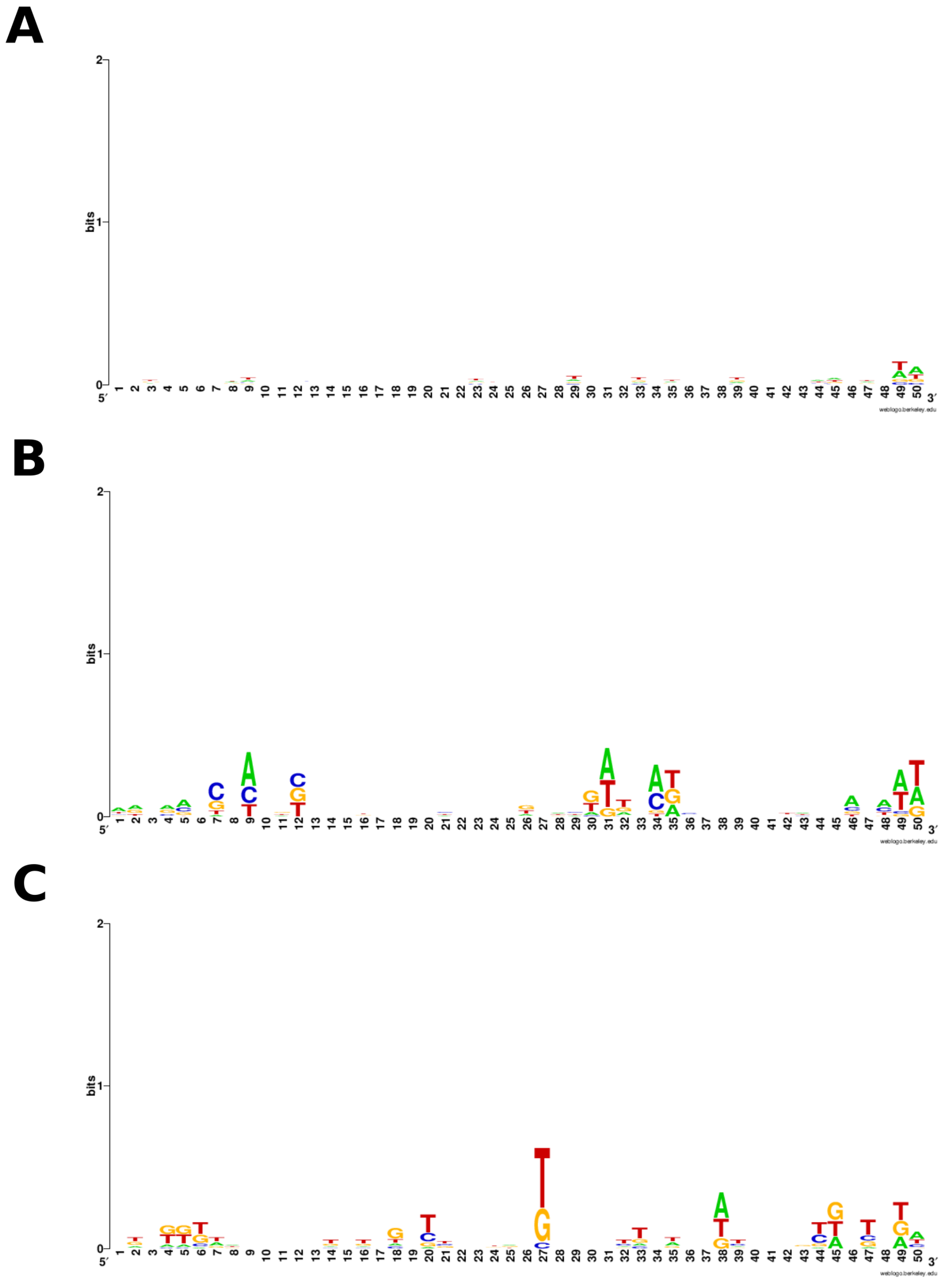

Supplementary Figure S8: Logo plots of sequences immediately upstream from start codons in Hodgkinia from Tettigades ulnaria. *Sequences were retrieved from either* ***(A)*** *all protein-coding genes,* ***(B)*** *the fifteen protein-coding genes with the highest average expression levels, or* ***(C)*** *the fifteen protein-coding genes with the lowest average expression levels.*

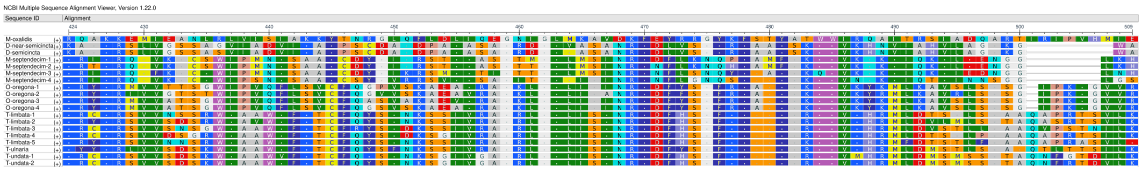

Supplementary Figure S9: Protein alignment showing deletions in domain 3 of the σ70 factor (RpoD) in some Hodgkinia lineages. *A portion of a protein alignment of all RpoD copies encoded by Hodgkinia lineages examined in this paper as well as RpoD from Hodgkinia in Diceroprocta semicincta and from the free-living alphaproteobacterium Methylobacterium oxalidis (retrieved from NCBI, protein accession: GEP04622.1) is shown. Hodgkinia from D. semicincta, D. near semicincta, and M. septendecim have gaps relative to other Hodgkinia lineages in the region spanning positions 501–507 of this amino acid alignment.*
